# 培美曲塞单药或联合铂类三线及以上治疗晚期非鳞型非小细胞肺癌的临床观察

**DOI:** 10.3779/j.issn.1009-3419.2012.02.10

**Published:** 2012-02-20

**Authors:** 飞 于, 晓晴 刘, 晓燕 李, 俭杰 李, 万峰 郭, 海峰 秦, 红军 高

**Affiliations:** 100071 北京，军事医学科学院附属医院肺部肿瘤科 Department of Pulmonary Oncology, Affiliated Hospital, Academy of Military Medical Sciences, Beijing 100071, China

**Keywords:** 培美曲塞, 肺肿瘤, 化疗, Pemetrexed, Lung neoplasms, Chemotherapy

## Abstract

**背景与目的:**

培美曲塞联合铂类或单药在晚期非小细胞肺癌一、二线治疗中的疗效已得到验证，但其在三线及以上治疗中的地位还不明确。本文旨在观察培美曲塞单药或联合铂类三线及以上治疗晚期非鳞型非小细胞肺癌的临床疗效及安全性。

**方法:**

46例多线治疗失败的晚期非鳞型非小细胞肺癌患者接受培美曲塞单药或联合铂类药物治疗。

**结果:**

46例晚期非鳞型非小细胞肺癌患者中部分缓解7例，疾病稳定20例，疾病进展19例，客观缓解率为15.2%，疾病控制率为58.7%，中位无疾病进展时间为3.0个月。分析显示培美曲塞联合卡铂及顺铂较培美曲塞单药治疗的疾病控制率明显增高（*P*=0.043）。常见的不良反应主要有恶心、呕吐及骨髓抑制。

**结论:**

应用培美曲塞单药或联合铂类治疗多线治疗失败的晚期非鳞型非小细胞肺癌患者仍可使其临床获益，且毒副反应可耐受。

## Abstract (en)

**Background and objective:**

Pemetrexed in combination with platinum or a single-agent has been approved for the first- and second-line treatment of non-small cell lung cancer (NSCLC). However, the role of pemetrexed therapy in the third-line and beyond treatment of NSCLC has yet to be generally accepted. The present retrospective study reports the efficacy and safety of pemetrexed in the third-line and beyond treatment of advanced non-squamous NSCLC.

**Methods:**

A total of 46 patients with advanced non-squamous NSCLC received a combination of pemetrexed plus platinum or a single-agent after multi-line treatment failed to yield positive results.

**Results:**

Of the 46 patients who participated in the study, 7 achieved partial responses, 20 reached a stage of stable disease, and 19 reached a stage of progressive disease. The over-all object response rate was 15.2% and the disease control rate (DCR) was 58.7%. The median progression-free survival time was 3.0 months. Pemetrexed in combination with platinum yielded a higher DCR than the pemetrexed plus single-agent treatment (*P*=0.043). Common adverse events included nausea, vomiting and myelosuppression.

**Conclusion:**

Administration of pemetrexed after failure of multi-line treatment is clinically beneficial to patients with advanced non-squamous NSCLC. The toxic effects of the treatment appear to be tolerable.

肺癌是全球发病率及死亡率最高的恶性肿瘤，非小细胞肺癌（non-small cell lung cancer, NSCLC）占肺癌的80%-85%，由于起病隐匿、发展迅速，2/3以上的患者临床确诊时已处于中晚期，失去了接受根治性手术的机会。第三代化疗药物联合铂类是晚期NSCLC一线治疗的标准方案，其客观缓解率为25%-35%，中位生存期为8个月-10个月^[1]^。一线治疗失败后约50%的患者有条件进入二线治疗，但二线治疗无论是化疗还是分子靶向治疗的客观缓解率都只有6.7%-9.1%，中位生存期仅5.5个月-8.3个月^[2-5]^。二线治疗失败后目前并没有标准三线化疗方案可供选择。2011年美国国家综合癌症网（national comprehensive cancer network, NCCN）指南对NSCLC患者三线治疗仅推荐分子靶向药物厄罗替尼。但对于部分二线治疗失败的患者来说，其一般情况较好，有进一步抗肿瘤治疗的必要，如何为这些患者选择治疗方案成为医生面临的难题。

培美曲塞是一种新型的多靶点抗叶酸制剂，与铂类药物联合应用是一线治疗晚期非鳞型NSCLC的标准方案；培美曲塞单药亦是晚期NSCLC二线治疗的推荐方案。但在实际临床工作中，因为用药选择、费用等种种原因，很多患者一线、二线治疗时未接受培美曲塞治疗，那么对有进一步治疗条件和治疗意愿的患者来说，三线及以上应用培美曲塞是否可以使患者临床获益以及治疗的耐受性都有待研究。本文回顾性分析了培美曲塞单药或联合铂类药物治疗三线及以上晚期非鳞型NSCLC的疗效及毒性反应，现报告如下。

## 1 材料与方法

### 1.1 病例选择

①患者均为组织学或细胞学证实的非鳞型NSCLC，包括肺腺癌、大细胞癌等；②根据国际TNM分期均为不可手术的Ⅲb期或Ⅳ期患者；③根据实体瘤评价标准（response evaluation criteria in solid tumors, RECIST），至少有1个可测量病灶；④美国东部肿瘤协作组（eastern cooperative oncology group, ECOG）评分≤2；⑤治疗前血常规、肝肾功、心脏功能基本正常，无其它严重疾病或合并症；⑥患者既往均经多线（≥2线）化疗或表皮生长因子受体酪氨酸激酶抑制剂（epidermal growth factor receptor tyrosinekinase inhibitor, EGFR-TKI）治疗失败；⑦既往未行培美曲塞化疗。

### 1.2 治疗方法

治疗方案包括：培美曲塞联合卡铂或顺铂和培美曲塞单药。用药方法：培美曲塞：500 mg/m^2^，第1天静脉滴注；首次培美曲塞给药前7天肌内注射维生素B12 1, 000 μg/次，每9周重复1次；首次培美曲塞给药前7天开始口服叶酸400 μg/d，持续至培美曲塞末次药物给予后3周；培美曲塞用药前日、当日和次日口服地塞米松8 mg/d。顺铂：75 mg/m^2^；或卡铂：AUC=5，第1天静脉滴注。化疗前给予5-HT3受体拮抗剂预防性止吐治疗，Ⅱ度以上度骨髓抑制者给予重组人粒细胞集落刺激因子治疗。每3周为1个治疗周期。肿瘤评估在基线、用药后每2周期进行。根据RECIST评价标准，达完全缓解（complete response, CR）、部分缓解（partial response, PR）或疾病稳定（stable disease, SD）的患者继续使用4周期-6周期。

### 1.3 评价标准

疗效评价按RECIST 1.1版标准判定为CR、PR、SD和疾病进展（progressive disease, PD）；客观缓解率（objective response rate, ORR）为CR+PR患者占全组患者的百分率；疾病控制率（disease control rate, DCR）为CR+PR+SD患者占全组患者的百分率；无疾病进展生存期（progression free survival, PFS）是指患者从首次用药至疾病进展或任何原因死亡的时间。

### 1.4 毒性反应

按照美国国家癌症研究所（national cancer institute, NCI）制定的毒性评价标准（第3版）评价毒性反应。

### 1.5 统计学方法

应用SPSS 13.0统计软件进行统计处理，疗效和临床特征的相关性分析采用χ^2^检验或*Fisher*确切概率法，PFS分析采用*Kaplan*-*Meier*曲线、*Cox*多因素生存分析模型。*P*＜0.05为差异有统计学意义。

## 2 结果

### 2.1 一般资料

2008年1月-2010年12月军事医学科学院附属医院肺部肿瘤科收治的晚期非鳞型NSCLC患者46例签署化疗知情同意书接受培美曲塞单药或联合铂类治疗，男性21例（45.7%），女性25例（54.3%）；治疗时中位年龄58岁（30岁-76岁），年龄≥60岁者20例（43.5%），年龄＜60岁者26例（56.5%）；病理类型为腺癌45例（97.8%），大细胞癌1例（2.2%）；吸烟者16例（34.8%），不吸烟者30例（65.2%）；体能状态（performance status, PS）评分0分-1分者45例（97.8%），2分者1例（2.2%）；临床分期Ⅲb期1例（2.2%），Ⅳ期45例（97.8%）；治疗时机：3线治疗29例（63.0%），3线以上治疗17例（37.0%）；用药方案：培美曲塞联合卡铂20例（43.5%），培美曲塞联合顺铂11例（23.9%），培美曲塞单药15例（32.6%）。距患者最近一次化疗的中位间隔时间为7个月。

### 2.2 疗效评价

46例晚期非鳞型NSCLC患者接受培美曲塞单药或联合治疗，中位治疗周期数为2周期（1周期-6周期）。所有患者均可评价疗效和毒性，无CR（0），PR 7例（7/46，15.2%），SD 20例（20/46，43.5%），PD 19例（19/46，41.3%），ORR为15.2%，DCR为58.7%。患者的临床特征与ORR及DCR的相关性见表 1。分析显示培美曲塞联合卡铂及顺铂治疗的DCR明显高于培美曲塞单药治疗（75%、63.34% *vs* 33.33%, *P*=0.043）。

**1:** 培美曲塞治疗晚期非鳞型NSCLC的临床特征与疗效的相关性 The relationship between the clinical characteristic and efficacy of pemetrexed in advanced non-squamous NSCLC

Characteristic	*n*	ORR [*n* (%)]	*P*	DCR [*n* (%)]	*P*
Gender			0.999		0.425
Male	21	3 (14.29)		11 (52.38)	
Female	25	4 (16.00)		16 (64.00)	
Age (year)			0.653		0.655
＜60	26	5 (19.23)		16 (61.54)	
≥60	20	2 (10.00)		11 (55.00)	
Smoking history			0.420		0.382
Ever smokers	16	1 (6.25)		19 (63.33)	
Never smokers	30	6 (20.00)		8 (50.00)	
Therapy			0.999		0.999
Third-line therapy	29	4 (13.79)		17 (58.62)	
Beyond third-line therapy	17	3 (17.65)		10 (58.82)	
Chemotherapy regimens			0.079		0.043
Pemetrexed	15	1 (6.67)		5 (33.33)	
Pemetrexed plus carboplatin	20	2 (10.00)		15 (75.00)	
Pemetrexed plus cisplatin	11	4 (36.36)		7 (63.34)	
ORR: objective response rate; DCR: disease control rate; NSCLC: non-small cell lung cancer.					

### 2.3 生存情况

数据收集截止时间为2010年12月，全组患者均随访至疾病进展，中位无疾病进展生存时间为3.0个月（1个月-7个月）（图 1），*Cox*多因素生存模型分析显示临床特征、用药方案、治疗时机等与PFS无明显相关性（表 2）。

**1:**
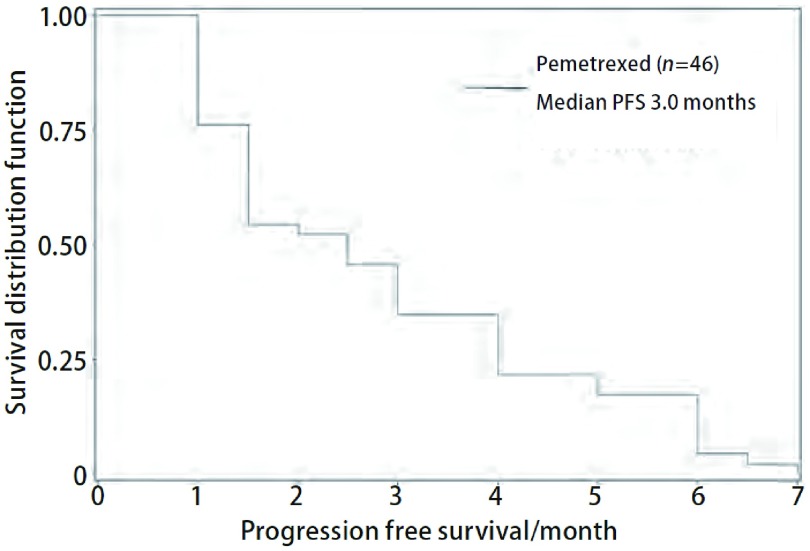
培美曲塞治疗46例非鳞型NSCLC患者无疾病进展生存曲线 *Kaplan*-*Meier* curves for PFS of pemetrexed in 46 non-squamous NSCLC patients

**2:** 培美曲塞治疗非鳞型NSCLC患者无进展生存期多变量*Cox*模型分析 The relationship between PFS and clinical chracteristic of non-squamous NSCLC patients analyzed by *Cox* multivariate model

Characteristic	*n*	RR_adjusted_	95%CI	*P*
Sex				
Male	21			
Female	25	1.54	0.39-6.15	0.573
Age (year)				
＜60	26			
≥60	20	0.98	0.35-2.74	0.850
Smoking history				
Ever smokers	16			
Never smokers	30	0.97	0.24-3.97	0.933
Therapy				
Third-line therapy	29			
Beyond third-line therapy	17	0.95	0.21-3.28	0.985
Chemotherapy regimen				
Pemetrexed	15			
Pemetrexed plus carboplatin	20	0.38	0.12-1.20	0.260
Pemetrexed plus cisplatin	11	0.52	0.14-1.91	0.660

### 2.4 毒副反应

全组毒副反应较轻，主要为骨髓抑制及胃肠反应，多为Ⅰ度至Ⅱ度，毒副反应达Ⅲ度至Ⅳ度主要有白细胞减少、血小板降低、贫血和恶心呕吐，发生率分别为15.3%、4.3%、2.2%、2.2%。总体不良反应轻微，均可耐受，对症处理后均能恢复正常，无治疗相关死亡（表 3）。

**3:** 培美曲塞治疗晚期非鳞型NSCLC的不良事件 The adverse events of pemetrexed in advanced non-squamous NSCLC

Adverse event	Ⅰ[*n* (%)]	Ⅱ [*n* (%)]	Ⅲ [*n* (%)]	Ⅳ [*n* (%)]
Leukopenia	5 (10.9)	5 (10.9)	4 (8.7)	3 (6.5)
Thrombocytopenia	1 (2.2)	2 (4.3)	2 (4.3)	0
Anemia	4 (8.7)	1 (2.2)	1 (2.2)	0
Nausea/Vomiting	6 (13.0)	5 (10.9)	1 (2.2)	0
Diarrhea	0	1 (2.2)	0	0
Fatigue	5 (10.9)	2 (4.3)	0	0
Febrile neutropenia	1 (2.2)	0	0	0
Aminotransferase, high	1 (2.2)	0	0	0

## 3 讨论

全身治疗是晚期NSCLC的主要治疗手段，以化疗、靶向治疗为主的综合治疗有助于延长晚期患者的生存期，提高生存质量。第三代化疗药物联合铂类方案是当前一线治疗晚期NSCLC的主要治疗方案。EGFR-TKI在晚期NSCLC治疗中也被证实有较好的临床获益，常用于二、三线治疗^[3, 6]^。2011年NCCN指南对NSCLC患者三线治疗仅推荐厄洛替尼单药、最佳支持治疗及参加新药临床试验等。但到目前为止，晚期NSCLC三线及以上治疗方案仍缺乏大型临床研究提供明确证据。对于大多数一般情况较好的患者，三线抗肿瘤治疗依然是必要及有效的，但参加新药临床试验的机会较少，因此三线及以上治疗面临困境。

培美曲塞是一种多靶点的叶酸拮抗剂，通过干扰细胞复制过程中叶酸代谢途径而发挥抗肿瘤作用，对包括NSCLC在内的多种肿瘤具有活性。其主要作用机制是抑制胸苷酸合成酶（thymidylate synthetase, TS）、二氢叶酸还原酶（dihydrofolate reductase, DHFR）和甘氨酸核糖核苷甲酰基转移酶（glycinamide ribonucleotide formyltransferase, GARFT），阻断嘧啶和嘌呤的合成，使细胞分裂停止在S期，从而抑制肿瘤细胞的生长^[7]^。

在培美曲塞对照多西他赛二线治疗晚期NSCLC的Ⅲ期临床研究（JMEI研究）中^[5]^，培美曲塞组中位生存期（overall survival, OS）为8.3个月，中位PFS为2.9个月，ORR为9.1%，与多西他赛组无统计学差异。而在非鳞癌亚组中，培美曲塞组则优于多西他赛组，OS分别为9.3个月和8.0个月（HR=0.778, 95%CI: 0.607-0.997），且培美曲塞组的安全性优于多西他赛。基于此项研究，美国食品和药物管理局（Food and Drug Administration, FDA）已于2004年批准培美曲塞为晚期NSCLC二线治疗药物。培美曲塞联合顺铂一线治疗NSCLC的Ⅲ期研究（JMDB研究）^[8]^显示培美曲塞联合铂类不劣效于第三代化疗药物联合铂类方案，且毒副作用较低，患者耐受性好。同样发现培美曲塞组在腺癌和大细胞癌中具有明显治疗优势，OS分别为11.8个月和10.4个月（*P*=0.005, HR=0.81, 95%CI: 0.70-0.94）。培美曲塞在NSCLC维持治疗方面的临床研究^[9]^也证实培美曲塞维持治疗相较于最佳支持治疗明显延长了PFS，尤其在非鳞癌亚组中，总生存期及无疾病进展生存期数据均显示培美曲塞组有较强的延长趋势。总之，培美曲塞在非鳞型NSCLC治疗中的作用与地位正逐步得到确认，临床应用逐渐广泛。但目前临床中，由于用药选择、治疗费用等种种原因，很多患者一线、二线治疗时未能接受培美曲塞治疗，因此三线及以上应用培美曲塞是否可以使患者临床获益及耐受性如何值得进一步探讨。

本文回顾性分析培美曲塞单药或联合铂类治疗46例非鳞型NSCLC患者的ORR为15.2%，DCR为58.7%，中位PFS为3.0个月，并且药物毒副作用较轻，大多数患者能够耐受。培美曲塞单药二线治疗晚期NSCLC临床研究（JMEI研究）^[5]^中，ORR为9.1%，中位PFS为2.9个月，中位OS为8.3个月。本研究中ORR略高于二线临床研究，而中位PFS与临床研究中培美曲塞二线治疗的结果相似，分析疗效略好的原因主要有本研究中所有患者均为非鳞型NSCLC患者；其次部分患者（占67.4%）的治疗方案为培美曲塞联合铂类治疗；最后大多数患者ECOG评分较好（PS评分0分-1分占97.8%），且女性（占54.3%）、不吸烟患者（占65.2%）较多。

虽然目前NSCLC二线及以上治疗的临床实践原则为单药治疗，但2009年发表的NSCLC二线双药对比单药治疗的*meta*分析^[10]^显示，患者二线双药治疗与单药治疗的ORR分别为15.1%和7.3%（*P*=0.000, 4），中位PFS分别为14周和11.7周（*P*=0.000, 9），都是双药优于单药，但两组的OS无差异（*P*=0.32），Ⅲ度-Ⅳ度血液学毒性和非血液学毒性是单药优于双药。因此，NSCLC二线双药与单药治疗比较虽然不能延长OS且毒副作用较大，但在ORR及中位PFS上仍有优势。此项研究拓展了我们的治疗思路。本研究中纳入的NSCLC患者PS评分大部分为0分-1分，化疗耐受性好，因此部分患者接受了培美曲塞联合铂类的双药治疗方案。分析结果显示培美曲塞联合卡铂、顺铂以及培美曲塞单药治疗非鳞型NSCLC的ORR分别为10%、36.36%和6.67%（*P*=0.079），统计学无明显差异，但培美曲塞联合铂类治疗疗效较培美曲塞单药有一定优势。而培美曲塞联合卡铂、顺铂以及培美曲塞单药治疗非鳞型NSCLC的DCR分别为75%、63.34%和33.33%（*P*=0.043），统计学具有明显差异，说明若患者一般情况好，能够耐受联合治疗，可能会在联合铂类的治疗中获益。

综上所述，培美曲塞作为一个新型的多靶点化疗药物，目前在NSCLC一线、二线治疗中的地位已得到确认。我们的结果初步表明对于多线治疗失败后的晚期非鳞型NSCLC患者，培美曲塞单药或联合铂类治疗亦可使部分患者获益，且安全性良好，但本文为回顾性临床研究且样本量小，结果仅作为临床参考，培美曲塞在三线及以上治疗中的确切疗效及安全性还需要更多前瞻性、大样本的临床研究来验证。

## References

[1] Waters JS, O'Brien ME (2002). The case for the introduction of new chemotherapy agents in the treatment of advanced non small cell lung cancer in the wake of the findings of The National Institute of Clinical Excellence (NICE). Br J Cancer.

[2] Fossella FV, DeVore R, Kerr RN (2000). Randomized phase Ⅲ trial of docetaxel versus vinorelbine or ifosfamide in patients with advanced non-small-cell lung cancer previously treated with platinum-containing chemotherapy regimens. The TAX 320 Non-Small Cell Lung Cancer Study Group. J Clin Oncol.

[3] Shepherd FA, Rodrigues Pereira J, Ciuleanu T (2005). Erlotinib in previously treated non-small-cell lung cancer. N Engl J Med.

[4] Thatcher N, Chang A, Parikh P (2005). Gefitinib plus best supportive care in previously treated patients with refractory advanced non-small-cell lung cancer: results from a randomised, placebo-controlled, multicentre study (Iressa Survival Evaluation in Lung Cancer). Lancet.

[5] Hanna N, Shepherd FA, Fossella FV (2004). Randomized phase Ⅲ trial of pemetrexed versus docetaxel in patients with non-small-cell lung cancer previously treated with chemotherapy. J Clin Oncol.

[6] Kim ES, Hirsh V, Mok T (2008). Gefitinib versus docetaxel in previously treated non-small-cell lung cancer (INTEREST): a randomised phase Ⅲ trial. Lancet.

[7] Molina JR, Adjei AA (2003). The role of pemetrexed (Alimta, LY231514) in lung cancer therapy. Clin Lung Cancer.

[8] Scagliotti GV, Parikh P, von Pawel J (2008). Phase Ⅲ study comparing cisplatin plus gemcitabine with cisplatin plus pemetrexed in chemotherapy-naive patients with advanced-stage non-small-cell lung cancer. J Clin Oncol.

[9] Ciuleanu T, Brodowicz T, Zielinski C (2009). Maintenance pemetrexed plus best supportive care versus placebo plus best supportive care for non-small-cell lung cancer: a randomised, double-blind, phase 3 study. Lancet.

[10] Di Maio M, Chiodini P, Georgoulias V (2009). *Meta*-analysis of single-agent chemotherapy compared with combination chemotherapy as second-line treatment of advanced non-small-cell lung cancer. J Clin Oncol.

